# Ascending aortic aneurysm after acute aortic dissection in a case with systemic lupus erythematosus

**DOI:** 10.1186/s44215-024-00165-3

**Published:** 2024-09-10

**Authors:** Hanae Sasaki, Ryosuke Kowatari, Yuki Imamura, Shintaro Goto, Akira Kurose, Masahito Minakawa

**Affiliations:** 1 Department of Thoracic and Cardiovascular Surgery, Hirosaki University School of Medicine, 5 Zaifu-cho, Hirosaki, Aomori, 036-8562 Japan; 2 Department of Pathology and Bioscience, Hirosaki University School of Medicine, Aomori, Japan; 3 Department of Anatomic Pathology, Hirosaki University School of Medicine, Aomori, Japan

**Keywords:** Systemic lupus erythematosus, Total arch replacement, Aortic aneurysm

## Abstract

A 50-year-old woman with systemic lupus erythematosus and a history of aortic arch replacement surgery for Stanford type A aortic dissection experienced a reoccurrence of an ascending aortic aneurysm and coronary artery occlusion. Computed tomography revealed that the aneurysm was compressing the superior vena cava and right atrium. The patient underwent urgent surgery to repair the aneurysm. This case highlights that aortic aneurysms can reoccur even after total arch replacement in systemic lupus erythematosus patients.

## Introduction

Systemic lupus erythematosus (SLE) is an autoimmune disease that predominantly affects women, often leading to vascular complications like aneurysms and dissections [1, 2]. This report focuses on a SLE patient who developed a true aortic aneurysm 17 years after a total arch replacement for acute aortic dissection (AAD).

## Case report

**Fig. 1:**
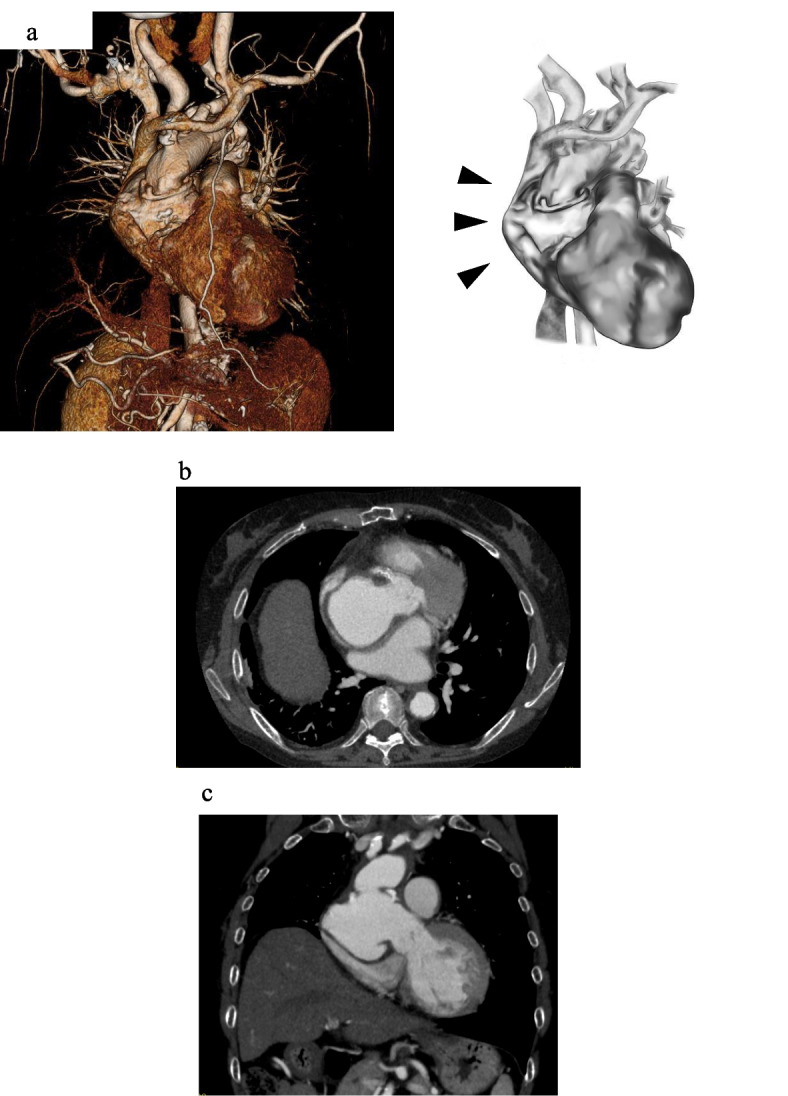
Computed tomography 3D reconstruction and its schema (**a**) showing the 63-mm ascending aorta near the proximal anastomosis site. The arrows indicate the aneurysm of the ascending aorta compressing the right atrium. The 2D axial view (**b**) and the 2D coronal view (**c**) display the aneurysm located between the previous surgery’s anastomosis site and the sinotubular junction D, dimensional

A 50-year-old woman, who has been managing systemic lupus erythematosus (SLE) with a daily dose of 4 mg prednisolone since age 18, experienced exertional dyspnea 17 years after undergoing an aortic arch replacement. Computed tomography (CT) showed a 63-mm ascending aortic aneurysm pressing on the superior vena cava and right atrium (Fig. 1). Cardiac catheterization identified a chronic total occlusion in the left anterior descending (LAD) artery. The patient underwent surgery for an ascending aortic aneurysm and occluded coronary artery. Considering the complexities of a reoperation and the long-term use of steroids, we opted for the saphenous vein graft (SVG) for the coronary artery bypass. Additionally, to ensure reliable myocardial protection, the SVG was deemed the most suitable choice for grafting to the LAD.

After median sternotomy and establishment of cardiopulmonary bypass (CPB), the harvested great saphenous vein was anastomosed to the LAD during systemic cooling to a bladder temperature of 23 °C. Cardioplegia was administered via the coronary ostium and the newly anastomosed saphenous vein graft. Subsequently, the aneurysmal section of the aorta was incised, the distal portion of the artificial blood vessel was clamped, and blood circulation was restored. The ascending aorta was replaced with a 24-mm J-graft (Japan Lifeline Co., Ltd., Tokyo, Japan), with proximal anastomosis performed at the sinotubular junction. The saphenous vein graft was then anastomosed to the J-graft, followed by aortic declamping. The durations of the surgical intervention, cardiac arrest, and circulatory arrest were 494, 113, and 3 min, respectively. No recurrence of the ascending aortic aneurysm was observed over a 2-year follow-up period.

**Fig. 2:**
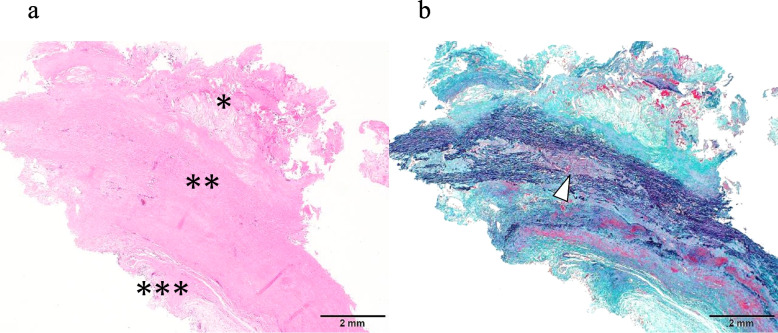
Histological findings of the aneurysm wall with severe atherosclerotic changes. **a** Hematoxylin and eosin (H&E) staining showing that the wall was composed of tunica intima (*), media (**), and externa (***). The tunica intima was thickened by the formation of an atheroma. **b** Masson–Goldner staining indicating the decrease of smooth muscle (red stained fibers) and the disordered arrangements of elastic fibers (brown to dark stained fibers). Some elastic fibers were ruptured, and fragmented fibers were observed (white arrow)

**Fig. 3:**
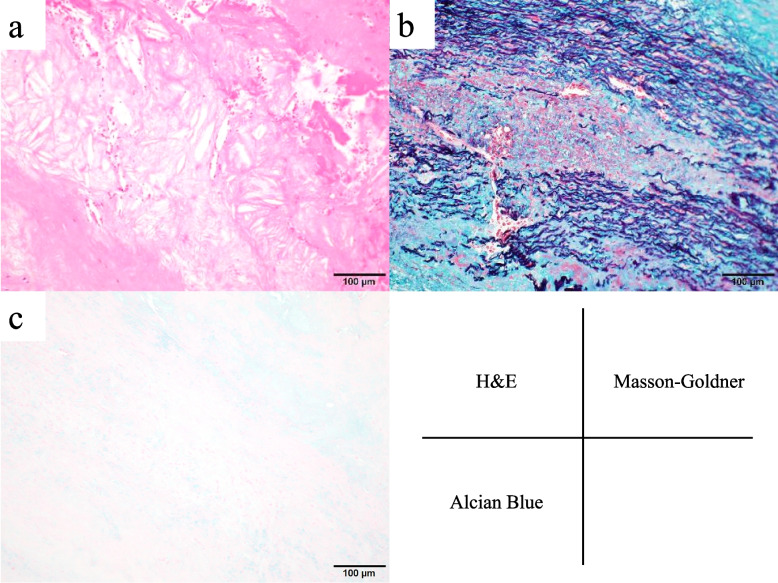
Higher-magnification image of the aneurysm wall. **a** Tunica intima showed advanced atheromatous changes with cholesterol clefts. **b** Masson–Goldner stain showed a cystic crevice between the elastic fibers of the tunica media. **c** Alcian blue stain did not show definite deposits of acidic mucopolysaccharide in the crevice of the tunica media

Histology confirmed it as a true aneurysm with pronounced atherosclerotic changes and potential cystic medial necrosis, despite the absence of clear mucopolysaccharide deposits (Figs. 2, 3).

## Discussion

Aortic dissections and aneurysms, while rare in the context of SLE, demonstrate a higher incidence in these patients compared to age- and sex-matched controls [1, 2]. Notably, SLE predisposes individuals to an earlier manifestation of aortic aneurysms [3]. These vascular anomalies are pathologically linked to cystic medial necrosis, characterized by the accumulation of mucopolysaccharides and Marfan-like alterations, including the deterioration of elastic fibers [4]. The histopathological findings in the present case, particularly the disruption of medial elastic fibers, suggest a potential role of such damage in the initial aortic dissection 17 years prior.

The etiology of aortic aneurysms in SLE extends beyond atherosclerosis to include mucoid degeneration, vascular trauma, hypertension, arteriosclerosis, and the impact of corticosteroid therapy [5]. Long-term corticosteroid administration is known to disrupt chondroitin sulfate synthesis and granulation tissue formation, thus compromising connective tissue integrity and heightening the risk of atherosclerosis [5]. Kurata et al. identified vasculitis and cystic medial degeneration as key factors in aortic aneurysm development in SLE patients, while atherosclerotic changes from prolonged corticosteroid use also play a significant role [6]. The present case exhibited a severe atherosclerotic TAA, with early onset and pronounced atherosclerotic lesions potentially attributable to SLE and sustained corticosteroid treatment. Although no direct evidence linked the initial aortic dissection to cystic medial necrosis, the International Registry of Acute Aortic Dissection reveals that individuals below 40 with aortic dissection often have underlying connective tissue disorders, suggesting a potential link in this case [7].

The comprehensive study on SLE-related aortic disease by Yuan and colleagues highlighted that aortic aneurysms comprised 67.5% of cases, dissections 27.5%, and concurrent aneurysms and dissections 5% [8]. The prevalence of such complications post-aortic surgery in SLE patients remains unclear due to SLE’s rarity. This case underscores the uniqueness of true aneurysm development in the residual proximal aortic wall long after aortic dissection surgery in an SLE patient. As evidenced by CT imaging, a significant issue in this instance was that too much of the ascending aorta was left intact during the initial operation. Had the entire ascending aorta wall been completely resected at that time, it is conceivable that the need for this reoperation might have been avoided. It is imperative for clinicians to recognize the potential for true aneurysm development in SLE patients even after arch replacement. Surgeons must be vigilant for the emergence of true aneurysms in SLE patients post-arch replacement and ensure no aortic wall segments are left at the aortic root. Given the absence of this specific pathology and its treatment strategy in current guidelines, diligent monitoring and the gathering of additional case reports are crucial for a deeper understanding of aortic dissections and aneurysms in the SLE population.

## Data Availability

Data will be made available on reasonable request.
